# From Implicit to Explicit: An Interaction-Reorganization Approach to Molecular Solvation Energy

**DOI:** 10.1021/acs.jctc.4c01283

**Published:** 2024-12-13

**Authors:** Kaifang Huang, Lili Duan, John Z.H. Zhang

**Affiliations:** † Shanghai Engineering Research Center of Molecular Therapeutics and New Drug Development, Shanghai Key Laboratory of Green Chemistry & Chemical Process, School of Chemistry and Molecular Engineering, East China Normal University, Shanghai 200062, China; ‡ School of Physics and Electronics, Shandong Normal University, Jinan, 250014, China; § Faculty of Synthetic Biology, Shenzhen University of Advanced Technology, Shenzhen 518055, China; ∥ Key Laboratory of Quantitative Synthetic Biology, Shenzhen Institute of Synthetic Biology, Shenzhen Institutes of Advanced Technology, Chinese Academy of Sciences, Shenzhen 518055, China; ⊥ NYU-ECNU Center for Computational Chemistry and Shanghai Frontiers Science Center of AI and DL, NYU Shanghai, Shanghai 200126, China; # Department of Chemistry, New York University, New York, New York 10003, United States

## Abstract

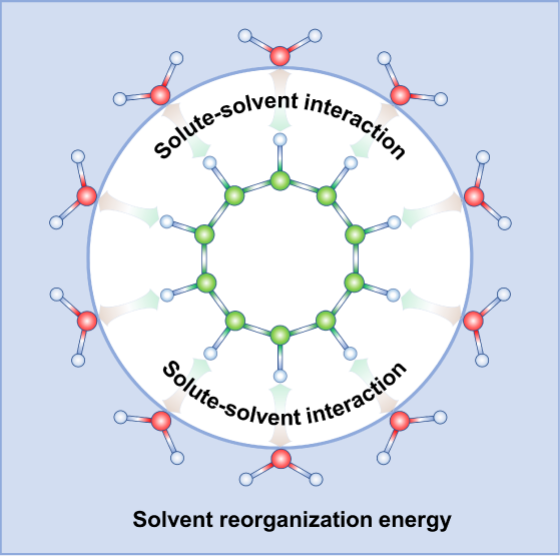

Accurate calculation of solvation energies has long fascinated researchers, but complex interactions within bulk water molecules pose significant challenges. Currently, molecular solvation energy calculations are mostly based on implicit solvent approximations in which the solvent molecules are treated as continuum dielectric media. However, the implicit solvent approach is not ideal because it lacks certain real solvation effects, such as that of the first solvation shell, etc. Here, we propose an explicit solvent approach, interaction-reorganization solvation (IRS) method, for molecular solvation energy calculations. The IRS approach achieves predictive accuracy comparable to that of the widely recognized solvation model based on the density (SMD) method and is significantly more accurate than that of the Poisson–Boltzmann/generalized Born surface area (PB/GBSA) methods. This is demonstrated in both the correlation coefficient and the mean absolute error (MAE) with respect to the experimental data. The IRS method is based on molecular dynamics simulation in explicit solvent and does not need to solve Poisson–Boltzmann or Schrödinger equations. On the other hand, the accuracy of the IRS method does depend on the accuracy of the molecular force field used in MD simulations. We expect that the IRS method will be very useful for the solvation energy calculations of molecules.

## 1 Introduction

Solvation effects play an indispensable role in physical, chemical, and biological processes. For example, in physical processes, solvation influences phase transitions, diffusion, and crystallization. In theoretical investigations of solution chemistry, the accurate determination of solvation free energies is essential for the assessment of reaction kinetics and the derivation of equilibrium constants for pertinent physical or chemical transformations.^1^ In biological processes, nearly all physiological activities occur in a solvent environment,^2^ affecting enzyme function, signal transduction, and molecular transport. Understanding these effects is vital for advancements in various scientific fields, including drug design, materials science, and environmental chemistry.

Given their importance, solvent effects have driven extensive research into predictive methodologies for solvation energies. Within the domain of quantum chemistry calculations, various continuum solvent models, including conductor-like screening model (COSMO),^3^ polarizable continuum model (PCM),^4^ solvation model based on density (SMD),^5^ and conductor-like polarizable continuum model (CPCM),^6,7^ are widely utilized. These models enable the precise and rapid prediction of solvation energies, which are essential for understanding solvent interactions and predicting molecular behavior in solution. Methods based on statistical mechanics and electrolyte theory, such as the Poisson–Boltzmann/generalized Born surface area (PB/GBSA)^8−17^ approaches, are particularly useful in the context of biomolecular systems, where they play an indispensable role in modeling solvent interactions and understanding the conformational dynamics of biomolecules. Recent advances in machine learning, such as ML-PCM,^1^ Delfos,^18^ and other models,^19−23^ have also contributed to advancements in accurately estimating molecular solvation free energies.

While these diverse methods offer robust tools for estimating solvation energies, it is crucial to recognize that the majority are based predominantly on continuum solvent models, which inherently possess limitations. Studies have shown that the protein-free energy landscapes predicted by these models differ significantly from those obtained using explicit solvent models.^24,25^ Notably, simple continuum models often fail to accurately represent critical nonpolar hydrophobic effects, which are important in biological processes occurring in water.^26,27^ Moreover, implicit solvent models tend to oversimplify predictions by neglecting the discrete nature of solvent–solute interactions at short distances, failing to provide a true molecular-level explanation of solvation effects.^28^ In contrast, atomistic simulation approaches in explicit solvent provide distinct advantages and yield detailed visualizations of the microscopic structures of the solvent shells surrounding solute molecules.^29^ These models deliver comprehensive physicochemical insights, revealing microscopic mechanisms of solvation and the complex interplay between solvent and solute molecules with the appropriate force fields and molecular dynamics parameters. This detailed approach offers a more nuanced understanding of solvation dynamics, which is essential for accurate simulations in computational chemistry. To address the gap between the capabilities of continuum and explicit solvent models, it is important to highlight the adaptability of free energy perturbation (FEP)^30−39^ and thermodynamic integration (TI)^30,40−46^ methods within explicit solvent frameworks. These alchemical methods calculate solvation energies by directly simulating each intermediate (alchemical) state in explicit solvent, offering a high degree of accuracy. However, their applicability is curtailed when dealing with larger molecules due to computational bottlenecks and convergence issues,^47,48^ which can severely limit their practical use in extensive or complex molecular systems. The Energy Representation (ER) method, developed by Matubayasi et al., characterizes solvent distribution functions through solute–solvent interactions to compute solvation energy,^49−52^ effectively predicting these energies for both small molecules^53^ and larger biomolecules such as proteins^54^ and polymers.^55^

In this work, we explore the utilization of explicit solvents to calculate the solvation energies of molecules. Our approach, termed the interaction-reorganization solvation (IRS) method, involves generating molecular dynamics simulations of the solute molecule in explicit solvent, thereby creating an extensive conformational space of solute conformations in solution. In this approach, the solvation free energy is decomposed into two terms, the solute–solvent interaction free energy and the reorganization free energy. The former is computed directly from MD simulations, while the latter is derived by fitting a polynomial expansion of the interaction term along with cavitation parameters from a training set that includes experimental solvation energies. Additionally, the interaction-reorganization solvation (IRS) method is tested on a validation set that does not overlap with the training data set, showing performance identical to that observed with the training set, thus confirming its efficacy in accurately calculating solvation energies.

## 2 Methods

### 2.1 Interaction-Reorganization Solvation (IRS) Method

**Figure 1:**
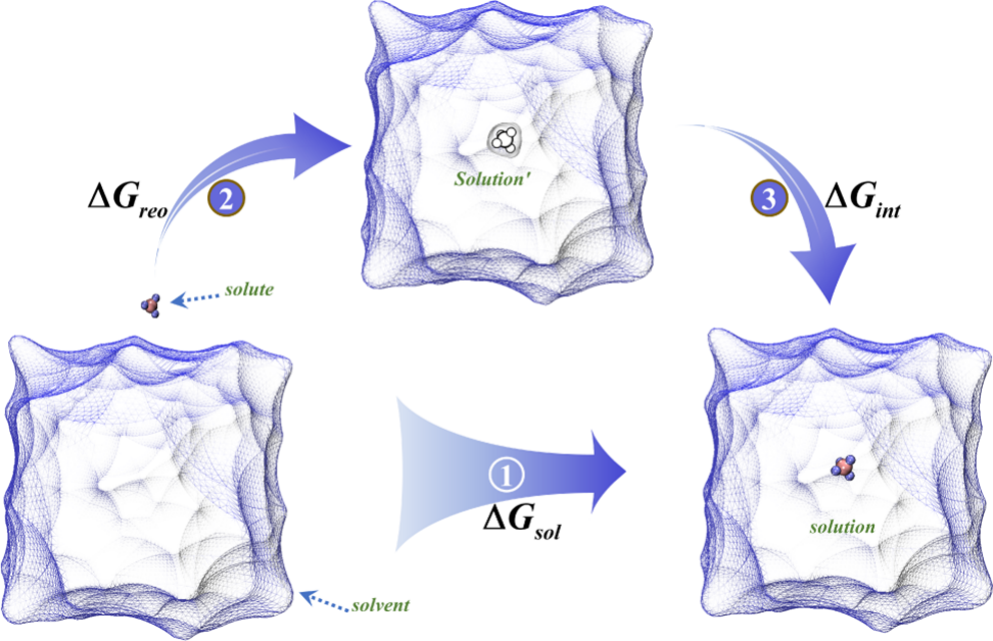
Thermodynamic cycle diagram illustrating the interaction-reorganization solvation method. Path 1 depicts the actual dissolution of the solute into the solvent, generating solvation energy. Paths 2 and 3 represent the computational stages within the IRS framework: Path 2 captures the formation of a solvent cavity by the solute, while Path 3 illustrates the subsequent interactions between the solute and solvent molecules.

As illustrated in Figure 1, the diagram depicts the thermodynamic cycle of a solute dissolved into a solvent to form a solution. From Path 1 in the diagram, according to the definition of Gibbs free energy, the solvation energy can be expressed as follows:
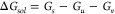
1

Where *G*_*s*_, *G*_*u*_, and *G*_*v*_ represent the Gibbs free energies of the solution (solvated system), the solute, and the solvent, respectively. According to the statistical mechanical definition, the Gibbs free energies of the components can be expressed in terms of their partition functions. Therefore, eq 1 can be reformulated as follows:
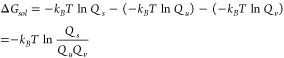
2

In the formula, *Q*_*s*_, *Q*_*u*_, and *Q*_*v*_ represent the partition functions of the solution, solute, and solvent, respectively. Each thermodynamic system partition function can be expressed in the following manner:
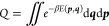
3

Where β = 1/*k*_*B*_*T* represents the inverse temperature factor with *k*_*B*_ as the Boltzmann constant and *T* is the temperature. Here, d***q*** and d***p*** represent the differential volume elements in the configuration space and momentum space, respectively. It is important to note that in eq 2, the contributions from the momentum integrals effectively cancel each other out. Therefore, in the subsequent derivation, we consider only the contributions from the internal energy. By substituting eq 3 into eq 2, we obtain
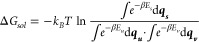
4

To facilitate the calculation of solvation energies, we modify the dissolution process by introducing a hypothetical state, denoted as *Q*_*s*_ (marked as *Solution’* in Figure 1). In this state, the solute enters the solvent and drains off the water molecules but does not interact with the solvent. This consideration simplifies the process by focusing solely on the exclusion of the solvent molecules without the involvement of additional interactions. Therefore, we revisit eq 1, which can be expressed as follows:
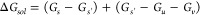
5

Similarly, eq 4 can be articulated as follows:
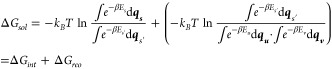
6

By referencing Figure 1, it is evident that the first term on the right-hand side of eq 6, denoted as Δ*G*_*int*_, corresponds to the energy change associated with Process 3, while the second term, Δ*G*_*reo*_, relates to the energy change from Process 2. We now proceed to derive each term to understand its contribution to the solvation process.

More thoroughly, eq 6 allows for the derivation of Δ*G*_*int*_ as follows:
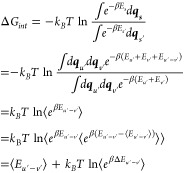
7

Here *E*_*u’*_, *E*_*v’*_, and *E*_*u’–v’*_ represent the internal energy of the solute and solvent, and the interaction energy between the solute and solvent, respectively. The ensemble average operator, ⟨···⟩, is evaluated by averaging over conformations from MD simulations. Eq 7^7^ illustrates that Process 3, depicted in Figure 1, exclusively involves the interaction energy between the solute and solvent, which is why this term is designated as the interaction energy, Δ*G*_*int*_. From our previous work,^56−58^ the interaction energy can be expressed in terms of the enthalpy–entropy relationship as follows:
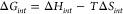
8

Comparing eq 7, the interaction enthalpy can be identified as
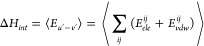
9

Here, under the Amber force field, the interactions between the solute and solvent (*E*_*u’–v’*_) can be characterized by the electrostatic (*E*_*ele*_) and van der Waals interactions (*E*_*vdw*_) between any two atoms, where *i* and *j* represent any atom of the solute and solvent molecules, respectively.

The interaction entropy is defined as
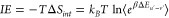
10which is positive definite, implying entropy loss resulting from the interaction. From the equations provided, it is evident that we can rapidly calculate the interaction energy between a solute and its surrounding solvent. Furthermore, the decomposition into enthalpy and entropy components enhances our understanding of the physical significance of solute–solvent interactions. This approach not only clarifies the nature of these interactions but also offers new insights into the dissolution mechanism, facilitating a deeper understanding of the underlying processes. Importantly, the energy component discussed is solely related to intermolecular interactions, allowing it to be computed in real-time during molecular dynamics simulations without incurring additional computational costs, with this efficiency ensuring that the method does not face bottlenecks due to computational resource constraints.

The second term in eq 6, Δ*G*_*reo*_, referred to as the solvent reorganization free energy arising from Process 2 illustrated in Figure 1, captures the energy change associated with a solute molecule entering the solvent, displacing solvent molecules, and inducing solvent reorganization under solute polarization to form a cavity. This term reflects the combined effects of polarization and cavitation energies due to the solute’s introduction into the solvent environment. In this study, the energy change due to solvent reorganization is approximated using two terms. The first term accounts for the energy associated with the displacement of solvent molecules by the solute, forming a cavity, which is quantitatively described by the “solvent accessible surface area (SASA)”. The second term addresses the energy change resulting from the reorganization of solvent molecules due to polarization and other interactions between the solute and solvent, which is assumed to be functionally equivalent to the interaction energy and represented as *f*(Δ*G*_*int*_). Thus, the solvent reorganization free energy can be approximated as
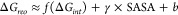
11where functional form of *f*(Δ*G*_*int*_) and fitting parameters γ and *b* will be determined in subsequent sections. The SASA is calculated using four common methods in Amber: solvent-accessible surface (SAS),^17^ solvent-accessible volume (SAV),^17^ linear combinations of pairwise overlaps (LCPO),^59^ and Molsurf.^60^

### 2.2 Data Set Preparation

We trained and validated our model using a standard data set. For the establishment of our data set, we utilized experimental solvation energy data for neutral molecules in water from the Minnesota Solvation Database (MNSOL)^61,62^ version 2012 as the benchmark. From an initial data set of 390 entries, 37 were excluded due to the presence of heavier halogens (Br and I), which exhibit distinct chemical behaviors compared to lighter halogens (F and Cl), or other complicating factors such as gaseous hydrogen, systems containing two water molecules, or the presence of a silicon atom. After applying these criteria, 353 systems remained, providing a focused and representative data set for our computational analysis.

The complete data set was randomly shuffled to ensure an unbiased distribution of samples. Subsequently, 280 systems were designated as the training set and employed to develop and refine our computational model. The remaining 73 systems were reserved as the test set, utilized to evaluate the model’s predictive accuracy and generalizability across unseen data.

### 2.3 Molecular Dynamics (MD) Simulation

The initial geometries of the solute molecules were retrieved from the MNSOL Database. These structures underwent an optimization process at the B97–3c^63^ level to refine their conformations. Charge distributions were then calculated using the ωB97M-V^64^ functional coupled with the def2-QZVP^65^ basis set, performed on the ORCA^66^ platform version 5.3.0. For charge fitting, we employed the Multiwfn^67^ software, specifically tailored to handle RESP2^68^ calculations.

Subsequent molecular dynamics simulations were initialized by immersing the optimized solute molecules in a truncated octahedral solvent model composed of TIP3P water molecules obtained by the tLEaP package, with a 15 Å buffer around each solute to simulate realistic environmental interactions. The optimization of solute–solvent assemblies was performed by using the steepest descent and conjugate gradient methods to minimize potential energy discrepancies. System equilibration was systematically carried out by heating to 298 K over 500 ps and then stabilizing the temperature and pressure during a 1 ns NPT equilibration phase, ensuring the system reached thermodynamic equilibrium. For accurate calculation of solvation energies, a 4 ns simulation period consisting of 10,000 frames was conducted to observe dynamic fluctuations and solute–solvent interactions. The MD simulation employed the General AMBER Force Field (GAFF)^69^ within the AMBER20^70^ software framework.

## 3 Results and Discussion

### 3.1 Interaction-Reorganization Solvation Free Energy

In order to elucidate the intricate nature of Δ*G*_*reo*_, this study utilizes an approach akin to a series expansion approach for *f*(Δ*G*_*int*_), as follows:
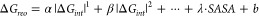
12where parameters α, β, ..., λ, and *b* in these equations are determined through multivariate linear regression, employing a training data set comprising experimental solvation energies. This approach allows for distinct formulations of Δ*G*_*sol*_ corresponding to various orders of expansion, delineated as follows:
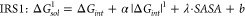
13
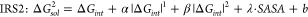
14
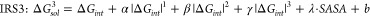
15
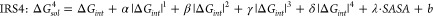
16

To avoid overfitting by introducing an excessive number of parameters, the expansion is limited to the fourth power. Considering the typical magnitude differences between interaction energies and solvation energies, such expansions could otherwise lead to the use of disproportionately large values to fit relatively small ones. For more nuanced modeling, a 3/2 power term is also explored:
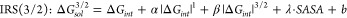
17

**Figure 2:**
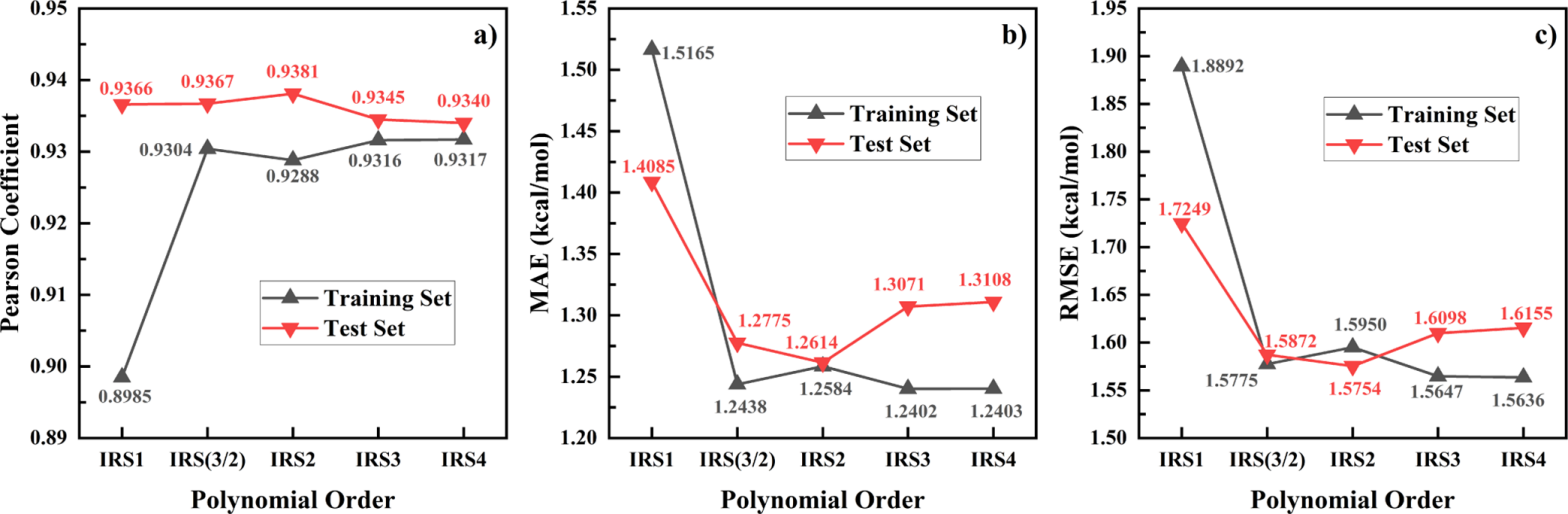
Performance of the IRS model across polynomial expansions in training and test sets. The *x*-axis represents various expansion orders as referred to eqs 13–17; panel (a) depicts Pearson correlation coefficients, panel (b) shows MAE, and panel (c) illustrates RMSE, with each metric presented for both the training and test sets.

To explore the efficacy of our approach, SASA was estimated using four different computational methods: SAS, SAV, LCPO, and Molsurf. The fitting results and the performance of these methods in both the training and test sets—measured by the Pearson correlation coefficient, Mean Absolute Error (MAE), and Root Mean Square Error (RMSE) between computed and experimental solvation energies—are detailed in Tables S1–S4. To concisely evaluate the computational capabilities of various polynomial expansions for calculating solvation energies, the average performance across four SASA calculation strategies serves as a representative measure, which is illustrated in Figure 2. In the training set, the Pearson correlation coefficient generally increases with the polynomial order, while RMSE and MAE decrease, suggesting that higher order expansions improve the IRS model’s accuracy in predicting solvation energies. However, in the test set, we observe a decline in the Pearson correlation coefficient and increases in MAE and RMSE when expanding beyond the second-order polynomial. This indicates that higher polynomial orders might lead to overfitting, corroborating our initial conjecture. Thus, considering the performance in both training and test sets, we recommend employing the IRS(3/2) and IRS2 models for a balanced trade-off between accuracy and generalization. In this work, unless specified otherwise, the IRS(3/2) model is the selected method for all subsequent discussions and calculations.

### 3.2 Performance on a Test Set

#### 3.2.1 Analysis of the IRS Model

**Figure 3:**
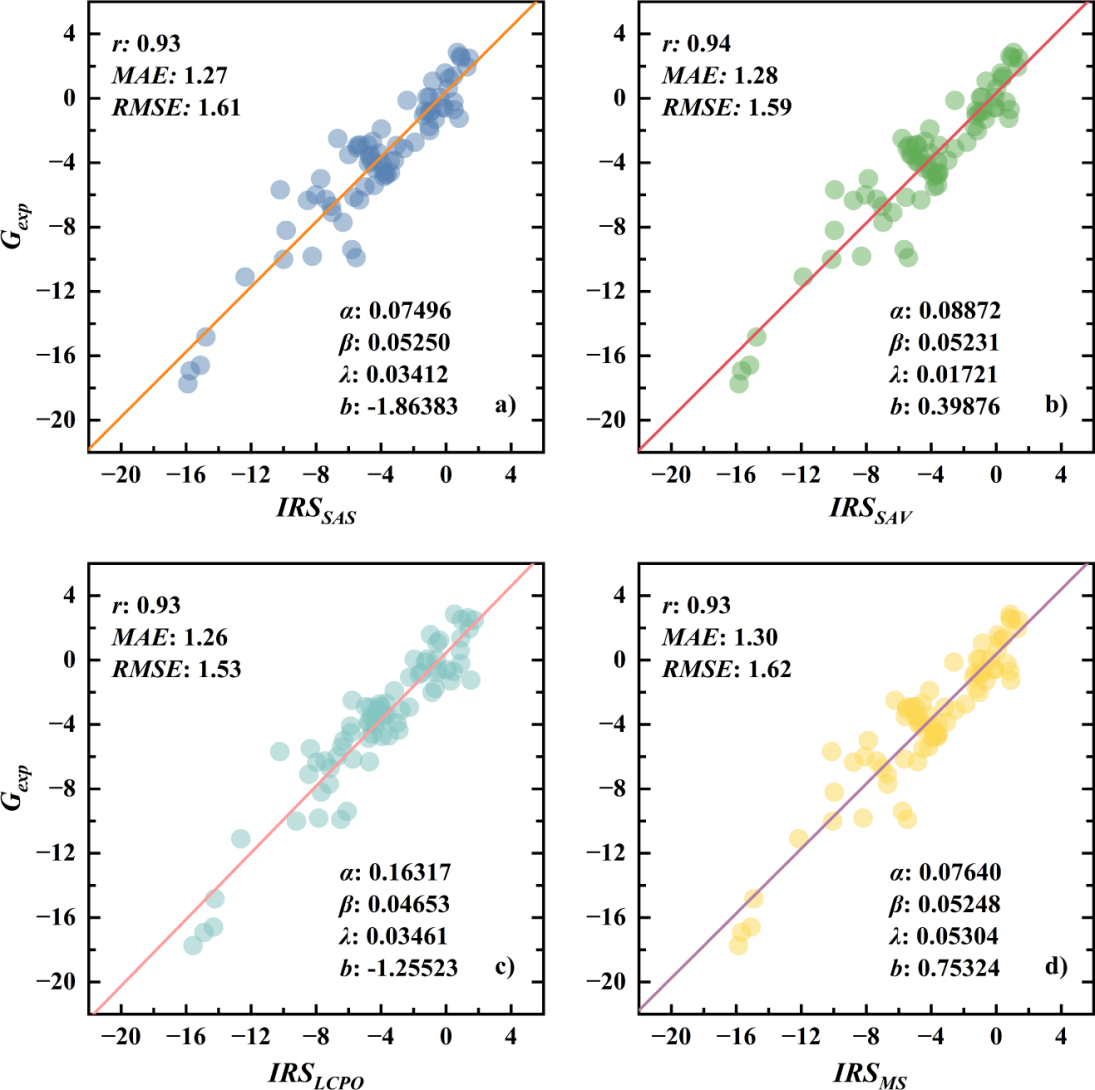
Scatter plots demonstrating the correlation between experimental and computed solvation energies for IRS in the test set. IRS models include IRS_SAS_ (a), IRS_SAV_ (b), IRS_LCPO_ (c), and IRS_MS_ (d); parameters displayed include Pearson correlation coefficient (*r*), mean absolute error (MAE), root mean square error (RMSE), and the fitting coefficients α, β, γ, and b as derived from eq 17. All energies are expressed in kcal/mol.

To ascertain the generalizability of the findings, the IRS model was employed on a test set comprising 73 diverse systems, which were entirely distinct from those in the training set. Reorganization energy, a component of solvation energy, was estimated by using four different solvent-accessible surface area (SASA) methodologies: SAS, SAV, LCPO, and Molsurf. These methods, denoted respectively as IRS_SAS_, IRS_SAV_, IRS_LCPO_, and IRS_MS_, were applied to estimate cavitation energy, with detailed fitting results and performance of these methods available in Figure 3. Initial analyses demonstrate that calculated solvation energies closely align with experimental values, as evidenced by scatter plots converging tightly around regression lines with slopes approaching unity and a Pearson correlation coefficient of 0.93. This strong alignment underscores a robust correlation with the experimental data. For the four IRS models, the MAE is approximately 1.3 kcal/mol, while the RMSE averages 1.6 kcal/mol, which affirms the precision of the IRS model in accurately predicting solvation energies. Furthermore, the analysis showed minimal variation in solvation energy predictions across different SASA methodologies, with Pearson coefficients ranging from 0.93 to 0.94, MAE spanning from 1.26 to 1.30, and RMSE from 1.53 to 1.62. This indicates that the IRS model’s predictions are not highly sensitive to the choice of SASA methodology, supporting the decision to average performances across these strategies as a representative outcome for the IRS method in the previous section.

However, there are some differences in the fitting parameters associated with different SASA methodologies. The α and β parameters in the expansion of Δ*G*_*int*_ are remarkably consistent across IRS_SAS_, IRS_SAV_, and IRS_MS_ methods but are somewhat different from those in the IRS_LCPO _method. To understand this disparity, we examined the correlations among SASA values computed by the four SASA methods in the training set. As shown in Table S5, the SASA values in SAS, SAV, and Molsurf methods are highly correlated (with correlation coefficients close to the unit), but correlations of these values from the LCPO method are only about 0.94 with any of the other three SASA methods. Given that reorganization solvation free energy is derived from a coupling of Δ*G*_*int*_ and SASA, this explains the difference of these two parameters in the LCPO method from those of the other three SASA methods.

To delineate the advantages of the IRS model over existing solvation energy prediction methods, Subsequent sections will highlight the IRS model’s computational accuracy over traditional implicit solvation models like PB/GBSA and SMD, and unless specified otherwise, will exclusively reference results from the IRS_SAV_ strategy.

#### 3.2.2 Comparison of IRS with PB/GBSA Models

**Figure 4:**
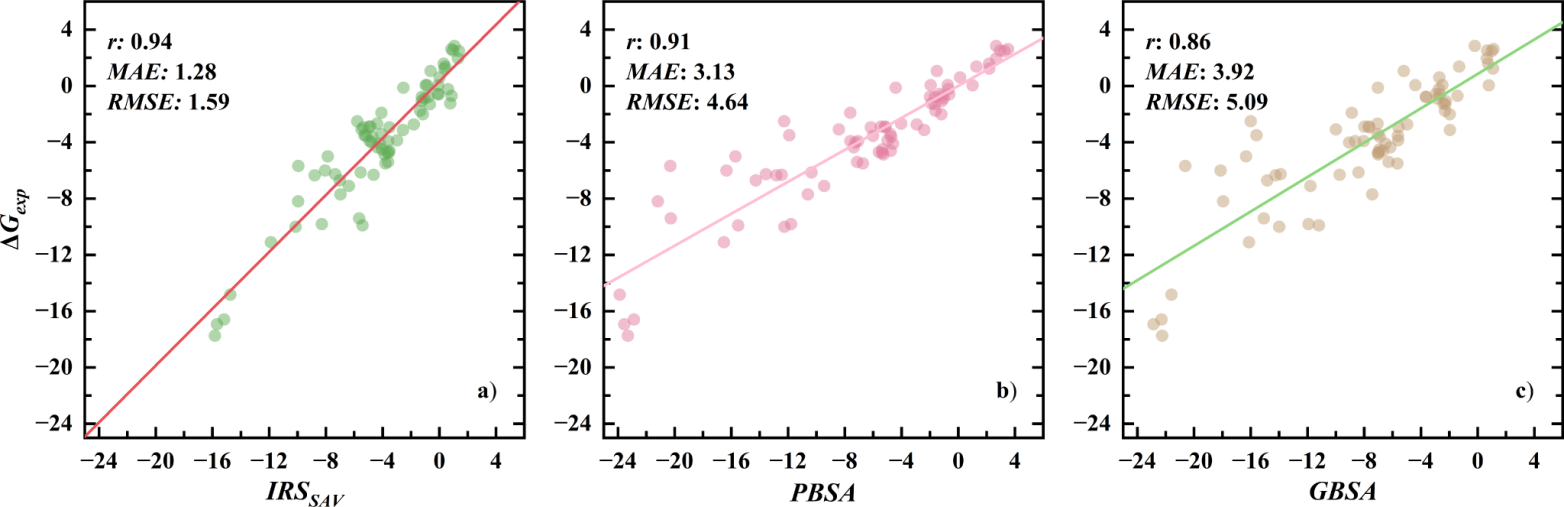
Scatter plots and performance metrics of solvation energy predictions for IRS_SAV_(a), PBSA(b), and GBSA(c) in the test set. All energies are expressed in kcal/mol.

Initially, comparing IRS with PB/GBSA—both methods that derive their averaged results from numerous conformations generated by force fields—the distinctions between the two are prominently highlighted in Figure 4. This comparison leverages the MMPBSA.py^71^ program’s default settings to evaluate both PB/GBSA methodologies. Specifically, for the PBSA calculations, polar solvation energies are computed using the Poisson–Boltzmann equation,^72,73^ and nonpolar terms are addressed via the solvent accessible volume (SAV)^17^ mode, which adopts a cavity surface tension of 0.0378 kcal/(mol·Å^3^) and an offset of −0.5692 kcal/mol. In parallel, the GBSA (generalized Born with surface area) approach employs the GB^OBC^ model II for polar energies,^16^ utilizing the LCPO method for nonpolar contributions, set with a surface tension of 0.005 kcal/(mol·Å^2^). The scatter plots demonstrate that the data points from the IRS method closely align with the regression line, indicating a tighter clustering compared to the relatively dispersed distribution observed with PB/GBSA models, which suggests that IRS predictions of solvation energies exhibit a stronger linear correlation with experimental values than PB/GBSA. Specifically, PB/GBSA models exhibit overall correlation coefficients of 0.91 for PBSA and 0.86 for GBSA—respectively high values—yet IRS surpasses these with a coefficient of 0.94, signaling more consistent alignment with experimental solvation energies. Moreover, the MAE and RMSE for IRS, at 1.28 and 1.59 kcal/mol, respectively, are almost one-third of those observed with PBSA and even lower when compared to GBSA, highlighting the enhanced accuracy of IRS in predicting solvation energies. Additionally, the slope of the regression line for PB/GBSA, approximately 0.6, indicates a general overestimation of solvation energies, a trend consistently observed in previous studies.^74,75^ In contrast, the IRS regression line has a slope of 1, demonstrating a very consistent match with experimental values even for a wider range of solvation energies, whose consistency underscores the model’s accuracy and its robust applicability across diverse molecular environments.

#### 3.2.3 Comparison of IRS and SMD Models

**Figure 5:**
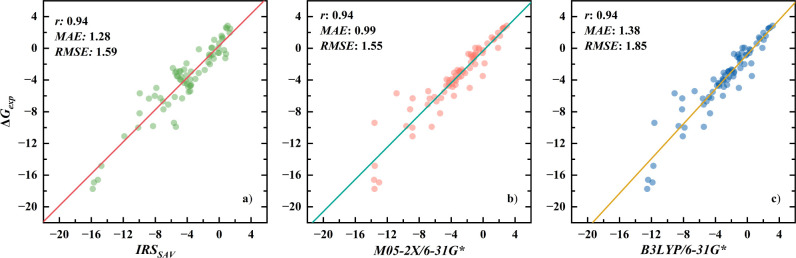
Scatter plots of solvation energy predictions against experimental values for the IRS_SAV_ model (a), and SMD models at M05-2*X*/6-31G* (b) and B3LYP/6-31G* (c) levels.^19^ All energies are expressed in kcal/mol.

Following the examination of IRS, a comparison was made with the solvation model based on density (SMD),^5^ a quantum mechanics method recognized for its high accuracy in predicting solvation energies of small molecules. Performance metrics for SMD were evaluated at the highly recommended M05-2*X*/6-31G*^71^ level by its developers.^5^ Considering the passage of over a decade since this endorsement, additional tests were performed using the same basis set but varying functional levels, specifically BLYP/6-31G*, to ensure a balanced assessment.^19^ These comparisons, as illustrated in Figure 5, demonstrate a precise alignment of scatter points along the regression lines for both IRS and SMD models, emphasizing their robust ability to accurately predict solvation energies—a consistency validated by a Pearson correlation coefficient of 0.94 across all strategies. In terms of precision, IRS and SMD exhibited similar performance at the M05-2*X*/6-31G* level, with some differences in mean absolute error (MAE), 1.28 for IRS versus 0.99 for SMD, while their root mean square errors (RMSE) were closely matched, 1.59 versus 1.55, respectively. This performance slightly surpasses that of SMD at the BLYP/6-31G* level, which reported an MAE of 1.38 and an RMSE of 1.85 kcal/mol. Moreover, similar to the PB/GBSA method, the regression line’s slope for SMD is not equal to 1, suggesting potential significant deviations from experimental values in systems with exceptionally high or low solvation energies. A detailed examination of the scatter plot distributions for IRS and SMD reveals a specific pattern: while SMD points tightly cluster around the regression line at lower solvation energies, they tend to deviate more at higher energy levels. This behavior indicates that although IRS may slightly underperform compared to SMD at lower energies, it markedly exceeds SMD’s accuracy at higher solvation energies.

**Figure 6:**
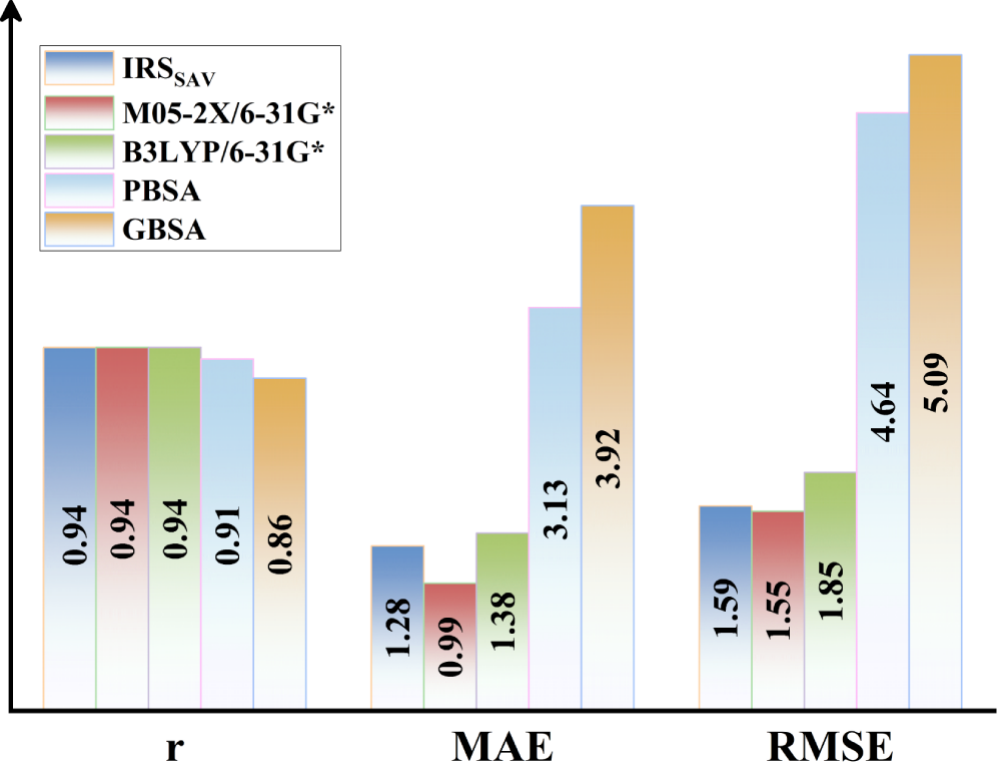
Comparative performance of IRS, SMD, and PB/GBSA in the training set, showcasing Pearson correlation coefficients (*r*), MAE, and RMSE. Pearson coefficients are emphasized with an enlarged bar representation for enhanced visibility.

For a clearer visual representation of the accuracy in predicting solvation energies, Figure 6 displays the correlation coefficients, MAE, and RMSE of the IRS, SMD, and PB/GBSA methods. This figure highlights the superior performance of IRS compared to PB/GBSA and its close alignment with the results from the SMD method. These comparative analyses reveal that IRS exhibits exceptional accuracy in estimating solvation energies for molecules, surpassing the widely used PB/GBSA models in biomolecular systems and achieving results comparable to those of the SMD method, thus positioning it as a competitive alternative in the field of solvation energy calculations.

### 3.3 Performance on the Entire Data Set

This section evaluates the comprehensive performance of the Interaction-Reorganization Solvation (IRS) model across the entire data set, encompassing both training and test sets. Our analysis integrates a wide spectrum of molecular systems, providing a robust assessment of the model’s predictive accuracy and reliability. Employing methods similar to those described in the previous section, our approaches were used to calculate SASA for IRS solvation energy calculations. For comparative purposes, PB/GBSA models and two different levels of quantum mechanical (QM) calculations with the SMD model were also utilized to predict solvation energies for this data set. The computational outcomes from all applied methodologies are comprehensively documented in Tables S1 and S3, with key performance metrics summarized in Table 1.

**Table 1:** Solvation Energy Calculation Performance for IRS, PB/GBSA, and SMD in the Entire Dataset

				SMD	
Method	IRS_SAV_	PBSA	GBSA	M05-2*X*/6-31G*	B3LYP/6–31G*^a^
*r*	0.94	0.90	0.86	0.93	0.93
MAE	1.22	3.57	3.97	1.03	1.38
RMSE	1.54	5.22	5.06	1.76	1.89

a Ref. (19), and *r* is Pearson correlation coefficient. All energy units are expressed in kcal/mol.

The analysis reveals a Pearson correlation coefficient of 0.94 for IRS in the entire data set, superior to the 0.90 and 0.86 observed with PBSA and GBSA, respectively. Furthermore, the MAE and RMSE for IRS, specifically 1.22 and 1.54 kcal/mol, respectively, are again approximately one-third those of PB/GBSA, mirroring the pattern established in the test set. This repeated performance demonstrates IRS’s consistent and superior predictive accuracy over PB/GBSA across different data sets. In the comparison with SMD, the trends observed during the test phase were replicated in the entire data set. Overall, the predictive capabilities of IRS for solvation energies closely align with those of SMD. Moreover, the transition from test to entire data sets showcased notable stability in IRS model performance. Specifically, the Pearson correlation coefficient remained stable, and the variations in MAE and RMSE were minimal, changing from 1.28 to 1.22 and 1.59 to 1.54 kcal/mol, respectively. The consistent recurrence of these performance patterns across various data sets not only confirms the high precision of the IRS model in predicting solvation energies but also highlights its robustness and reliability across computational contexts.

Noteworthy is that, despite its unique advantages in computational precision, the SMD is limited in application to small molecules due to its reliance on quantum mechanical calculations of electron density functions. On the other hand, the PBSA and GBSA methods, although less precise with a larger error margin, maintain a consistent Pearson correlation coefficient around 0.90 and 0.86, respectively. This robustness allows for a reasonably accurate description of solvation energy effects within a computationally feasible framework, making it a prevalent choice for solvation energy calculations in biomolecular systems. The IRS method introduced in this work primarily addresses the issue of computational demand. IRS calculations, which involve solute–solvent interaction energies and SASA, benefit from the efficiencies in the MD simulation processes. The interaction energies, theoretically precomputed during MD simulations, can be concurrently output, while the computational resources required for SASA calculations are minimal. On the contrary, the accuracy of IRS in predicting solvation energies has been rigorously validated across both test and entire data sets, showing performance comparable to the SMD benchmarks recommended at the M05-2*X*/6-31G* level. Therefore, IRS not only alleviates computational burdens but also maintains high accuracy in solvation energy predictions, offering a compelling alternative for extensive molecular system analyses.

Furthermore, we observed an intriguing inconsistency in the SMD method concerning two critical metrics used to evaluate our novel approach: MAE and RMSE. Although these metrics are not linearly correlated from a mathematical standpoint, they typically exhibit synchronous behavior when assessing data stability, where larger MAE values generally align with larger RMSE values and vice versa, a trend that was generally observed in this work. However, this expected pattern did not hold in several cases within the SMD evaluations. More precisely, within the test set, the SMD at the M05-2*X*/6-31G* level recorded the lowest MAEs, underperforming all of the IRS strategies by at least 0.27 kcal/mol. Despite this, the RMSE for SMD closely matched that of IRS, with the largest discrepancy being a mere 0.07 kcal/mol. Across the entire data set, the disparity was even more marked; SMD’s MAE at the M05-2*X*/6-31G* level was significantly lower than IRS_SAV_ (1.03 vs 1.22 kcal/mol), yet its RMSE was 0.22 kcal/mol higher. This discrepancy suggests that while the SMD method can predict solvation energies for some molecules with high precision, it exhibits significant errors for others, possibly leading to observed deviations from experimental values. This hypothesis is explored in more detail in the following sections of this work.

### 3.4 Error Analysis

**Figure 7:**
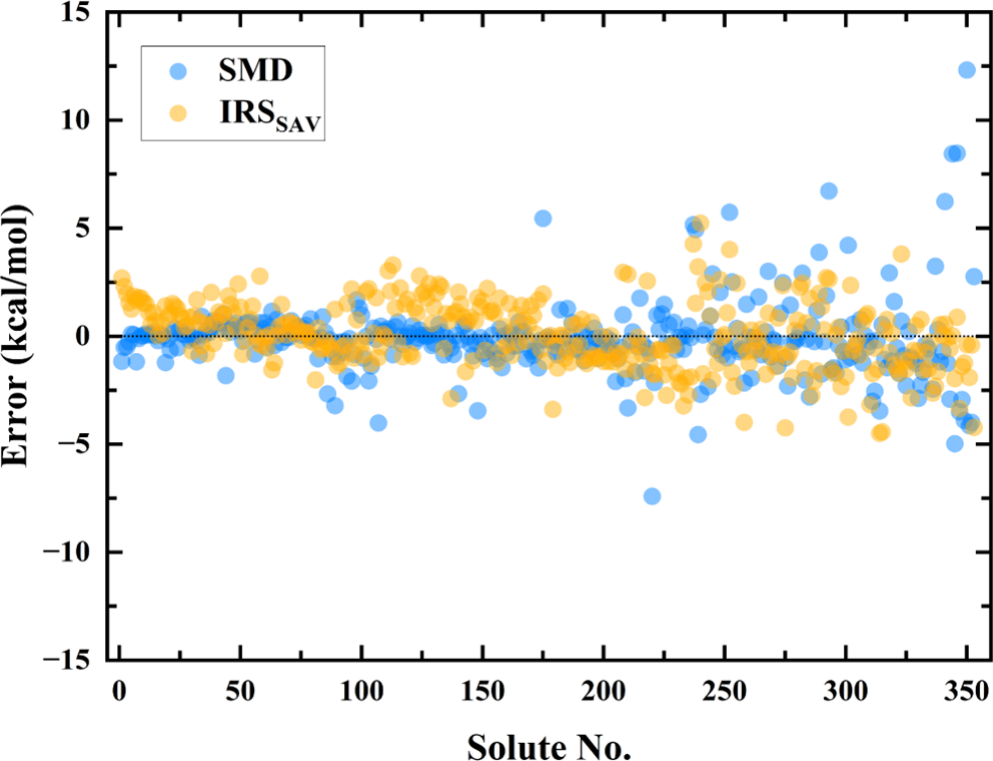
Error distribution between experimental and predicted solvation energies by IRS and SMD (at the M05-2*X*/6-31G* level) methods. The vertical axis shows the error, defined as the difference between experimental and predicted solvation energies, while the horizontal axis is indexed by solute, sorted by experimental solvation energy, as detailed in Table S6. The dotted line represents the zero-reference line, indicating no deviation from experimental values.

To elucidate the origins of computational discrepancies of IRS method and examine the unusual MAE and RMSE synchronicity observed in SMD methodologies, deviations between computed solvation energies from IRS and SMD and their corresponding experimental values were analyzed across a data set sorted by experimental solvation energies, encompassing both training and test sets. Here, to clearly illustrate the patterns, we selected the best-performing strategies for both the IRS and SMD, specifically IRS_SAV_ and M05-2*X*/6-31G*, as depicted in Figure 7. As illustrated, the SMD error tends to increase with solvation energy, a trend corroborated by the alignment of SMD data points along the regression line at lower energies and significant deviations at higher energies seen in Figure 5b. Although IRS exhibits a similar trend, its deviation is notably less pronounced, supporting its broader applicability across varying solvation energies. This observation was also anticipated in Section 3.2, where the slope of the regression line for IRS predictions of solvation energies was determined to be 1, indicating consistent accuracy across a wide spectrum of solvation energy. Furthermore, the consistent occurrence of outliers in SMD calculations, regardless of the solvation energy, accounts for the observed lower MAE but higher RMSE, suggesting that SMD is selectively sensitive to specific molecular configurations. In contrast, the IRS displays substantial robustness and a broader universality, showing less sensitivity to variations in molecular systems. Such differences may stem from the foundational methodologies: SMD relies on single-conformation-based solvation energy calculations, whereas IRS calculates an ensemble average. The comparative analysis underscores that while SMD shows selective sensitivity, impacting its reliability across diverse molecules, IRS maintains a high level of accuracy and robustness. This substantiates IRS’s superiority in consistently predicting solvation energies across a broader range of molecules. The consistent and reliable performance of IRS highlights its potential as a universally applicable method in the computational estimation of solvation energies, positioning it as a robust tool for theoretical and practical applications in computational chemistry.

### 3.5 Entropy Contribution to Solvation Free Energy

**Figure 8:**
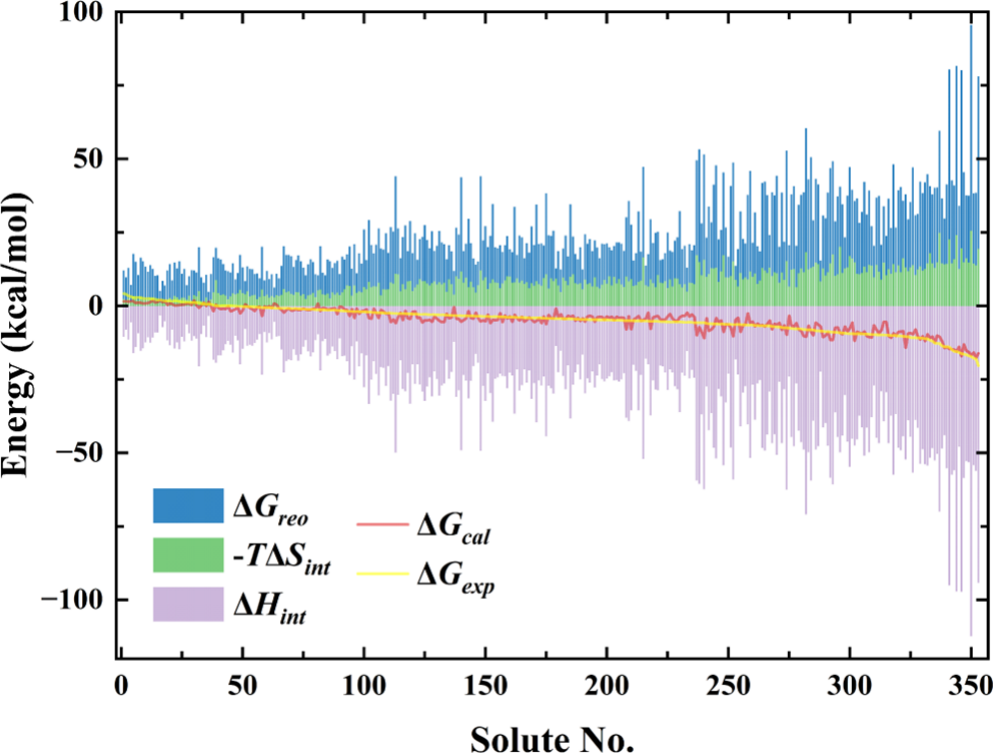
Stacked column chart of solvation energy components calculated using the IRS_SAV_ method. the red line represents the experimental solvation energies (Δ*G*_*exp*_), and the yellow line indicates the calculated solvation energies (Δ*G*_*cal*_) by IRS_SAV_. The horizontal axis, labeled “Solute No.”, indexes systems sorted by increasing experimental solvation energy as shown in Table S6.

A distinct advantage of the IRS is its capacity to elucidate the individual contributions of interaction enthalpy, interaction entropy, and cavitation energy to the overall solvation energy, thus providing a nuanced understanding of the solvation phenomena. In this study, we have illustrated these contributions using stacked column charts as shown in Figure 8. Visual analysis of the stacked column chart clearly indicates that interaction entropy and cavitation energy contribute positively, thus exerting adverse effects during the solvation process, whereas interaction enthalpy presents negative values, signifying its facilitative role in the process. This observation aligns with theoretical expectations: the introduction of a solute into a solvent typically requires energy to displace solvent molecules (an endothermic process), while the electrostatic and van der Waals interactions between the solute and solvent (interaction enthalpy) energetically favor solvation, albeit contributing to an increase in entropy. This multifaceted interplay highlights the complexity of solvation dynamics with each component playing a critical role in the overall energy balance. Notably, as the solvation energy increases, the contribution of entropy also grows, suggesting that entropy plays an increasingly significant role. In contrast, the SMD primarily considers solvation energies as contributions from bulk electrostatics and short-range interactions without directly accounting for entropy effects. This oversight may account for the observed underperformance of SMD in larger molecular systems compared to IRS, suggesting that IRS’s comprehensive approach to modeling all pertinent energy components offers a more accurate and reliable prediction of solvation energies, particularly in large and complex molecular systems.

## 4 Conclusion

In this work, we introduced a method for calculating solvation energies using explicit water molecules. the Interaction-Cavitation Solvation (IRS). This explicit method has some distinct features that are not present in the traditional implicit solvent models,

First, the IRS method provides an accurate representation of solvation effects, capturing the complex interactions between the solute and the solvent molecules. This realism is essential for applications involving intricate molecular systems, where the precise nature of solute–solvent interactions significantly impacts their behavior.

Second, the IRS method demonstrates superior performance compared to the PB/GBSA methods and achieves results comparable to those of the SMD method. For comparison, the IRS method does not need to solve the Poisson–Boltzmann equation in the PB method or Schrödinger equation in the SMD method.

Finally, the IRS framework elucidates the discrete contributions of different solvation components, furnishing novel insights into the solvation dynamics. Particularly, the analysis of solvation energy components through IRS underscores the pivotal role of entropy in the solvation processes of a molecule, highlighting its indispensable contribution that cannot be overlooked.

It should be noted that the IRS method derives its configurations and energy calculations from force fields, conferring a substantial theoretical advantage. Unlike the PBSA (Poisson–Boltzmann with surface area) method, which transitions from force-field-derived conformations to electrolyte theory, the IRS maintains methodological consistency throughout. However, this fidelity is a double-edged sword as IRS’s accuracy depends critically on the precision of the underlying force field. Additionally, due to the complexity of reorganization energy, this study approximates it using interaction energy and solvent-accessible surface area only, a simplification that necessitates exploring more rigorous ab initio methods to quantify this component accurately.

## Supporting Information Available

IRS fitting parameters, performance metrics for IRS method and SASA Correlation Matrix (PDF)
Solvation energy predictions and errors for IRS, PB/GBSA, and SMD across training and test sets; IRS energy components (XLSX)
(XLSX)
(XLSX
