# Supplementary material for: From Implicit to Explicit: An Interaction-Reorganization Approach to Molecular Solvation Energy
Source: J Chem Theory Comput. 2024 Dec 13;20(24):10961–71. doi: 10.1021/acs.jctc.4c01283 (PMC11674157; doi:10.1021/acs.jctc.4c01283)
Supplement: Supplementary file 1 — ct4c01283_si_001.pdf [file ct4c01283_si_001.pdf]

Supplementary Information

From Implicit to Explicit: An Interaction-Reorganization Approach to Molecular Solvation Energy

Kaifang huang[1], Lili Duan[2*], John Z.H. Zhang[1,3,4,5,6*]

1. *Shanghai Engineering Research Center of Molecular Therapeutics and New Drug Development, Shanghai Key Laboratory of Green Chemistry & Chemical Process, School of Chemistry and Molecular Engineering, East China Normal University, Shanghai 200062, China*
2. *School of Physics and Electronics, Shandong Normal University, Jinan, 250014, China*
3. *Faculty of Synthetic Biology, Shenzhen University of Advanced Technology, Shenzhen 518055, China.*
4. *Key Laboratory of Quantitative Synthetic Biology, Shenzhen Institute of Synthetic Biology, Shenzhen Institutes of Advanced Technology, Chinese Academy of Sciences, Shenzhen 518055, China*
5. *NYU-ECNU Center for Computational Chemistry and Shanghai Frontiers Science Center of AI and DL, NYU Shanghai, Shanghai 200126, China;*
6. *Department of Chemistry, New York University, New York, New York 10003, United States;*

*Correspondences to: duanll@sdnu.edu.cn, john.zhang@nyu.edu

Table S1. Solvation energy predictions by IRS across various polynomial orders (Eqs. 13-17), PB/GBSA, and SMD methods in the training set. All Energy in kcal/mol; Table S1 is provided as an Excel file named TableS1

Table S2. Fitting parameters for various polynomial expansions of IRS model derived from training set.

Order	Method	α	β	γ	δ	λ	b
IRS1	IRS_{SAS}	0.528734				0.030767	-4.626831
	IRS_{SAV}	0.537980				0.015663	-2.606563
	IRS_{LCPO}	0.555228				0.032819	-3.970107
	IRS_{MS}	0.529684				0.047885	-2.269118
IRS(3/2)	IRS_{SAS}	0.074958	0.052495			0.034115	-1.863830
	IRS_{SAV}	0.088724	0.052307			0.017211	0.398760
	IRS_{LCPO}	0.163170	0.046526			0.034608	-1.255227
	IRS_{MS}	0.076397	0.052479			0.053037	0.753238
IRS2	IRS_{SAS}	0.294494	0.003289			0.034156	-2.776161
	IRS_{SAV}	0.305222	0.003303			0.017280	-0.499192
	IRS_{LCPO}	0.360579	0.002878			0.034574	-2.080254
	IRS_{MS}	0.294070	0.003308			0.053224	-0.148976
IRS3	IRS_{SAS}	0.108860	0.009410	-0.000052		0.033827	-1.286793
	IRS_{SAV}	0.161065	0.008093	-0.000040		0.017068	0.665058
	IRS_{LCPO}	0.154475	0.009627	-0.000057		0.034344	-0.465244
	IRS_{MS}	0.139085	0.008456	-0.000043		0.052560	1.095443
IRS4	IRS_{SAS}	0.108799	0.009413	-0.000052	4.60×10^{-10}	0.033827	-1.286463
	IRS_{SAV}	0.147825	0.008838	-0.000056	9.88×10^{-08}	0.017066	0.735406
	IRS_{LCPO}	0.061327	0.014841	-0.000164	6.91×10^{-07}	0.034414	0.014860
	IRS_{MS}	0.133192	0.008788	-0.000050	4.40×10^{-08}	0.052557	1.126825

Table S3. Solvation energy calculated by IRS across various polynomial orders (Eqs. 13-17), PB/GBSA, and SMD methods in the test set. All Energy in kcal/mol; Table S3 is provided as an Excel file names TableS3.

Table S4: Performance metrics for IRS across various polynomial orders in training and test sets. Metrics included: Pearson Correlation Coefficient (r), Mean Absolute Error (MAE), and Root Mean Square Error (RMSE) with MAE and RMSE expressed in kcal/mol.

Metric		Training Set			Test Set		
Order	Method	r	MAE	RMSE	r	MAE	RMSE
IRS1	IRS_{SAS}	0.8941	1.5419	1.9285	0.9335	1.4407	1.7631
	IRS_{SAV}	0.9017	1.4864	1.8614	0.9351	1.4259	1.7337
	IRS_{LCPO}	0.9034	1.4994	1.8462	0.9442	1.3187	1.6435
	IRS_{MS}	0.8950	1.5384	1.9206	0.9335	1.4488	1.7592
	Average	0.8985	1.5165	1.8892	0.9366	1.4085	1.7249
IRS(3/2)	IRS_{SAS}	0.9278	1.2565	1.6063	0.9356	1.2677	1.6069
	IRS_{SAV}	0.9350	1.2099	1.5267	0.9359	1.2786	1.5884
	IRS_{LCPO}	0.9302	1.2484	1.5802	0.9413	1.2640	1.5335
	IRS_{MS}	0.9287	1.2604	1.5969	0.9341	1.2997	1.6199
	Average	0.9304	1.2438	1.5775	0.9367	1.2775	1.5872
IRS2	IRS_{SAS}	0.9260	1.2694	1.6250	0.9366	1.2569	1.5996
	IRS_{SAV}	0.9337	1.2226	1.5412	0.9374	1.2598	1.5742
	IRS_{LCPO}	0.9282	1.2704	1.6018	0.9428	1.2439	1.5212
	IRS_{MS}	0.9273	1.2714	1.6120	0.9355	1.2850	1.6066
	Average	0.9288	1.2584	1.5950	0.9381	1.2614	1.5754
IRS3	IRS_{SAS}	0.9292	1.2533	1.5911	0.9335	1.2957	1.6286
	IRS_{SAV}	0.9357	1.2088	1.5197	0.9345	1.3002	1.6027
	IRS_{LCPO}	0.9321	1.2422	1.5597	0.9377	1.3133	1.5720
	IRS_{MS}	0.9295	1.2566	1.5882	0.9324	1.3193	1.6358
	Average	0.9316	1.2402	1.5647	0.9345	1.3071	1.6098
IRS4	IRS_{SAS}	0.9292	1.2528	1.5909	0.9336	1.2952	1.6268
	IRS_{SAV}	0.9357	1.2100	1.5193	0.9342	1.3028	1.6064
	IRS_{LCPO}	0.9324	1.2408	1.5564	0.9359	1.3244	1.5899
	IRS_{MS}	0.9295	1.2576	1.5878	0.9322	1.3208	1.6388
	Average	0.9317	1.2403	1.5636	0.9340	1.3108	1.6155

Table S5. Pearson correlation coefficients among SASA calculation methods in the training set.

Pearson	SAS	SAV	LCPO	Molsurf
SAS	1.0000	0.9962	0.9393	0.9992
SAV	0.9962	1.0000	0.9396	0.9977
LCPO	0.9393	0.9396	1.0000	0.9367
Molsurf	0.9992	0.9977	0.9367	1.0000

Table S6. IRS energy components and prediction errors from IRS, PB/GBSA, and SMD methods across entire dataset, sorted by experimental solvation energies. All energy expressed in kcal/mol. Table S6 is provided as an Excel file names TableS6.

Table S7. Comparison of solvation energy predictions between IRS and ER Methods across various molecules. ΔG_{cal} and ΔG_{exp} represent calculated and experimental solvation energies, respectively; all energy units are kcal/mol.

FileHandle	Name	IRS_{SAV} ΔG_{cal}	ER $\Delta G_{cal}(\Delta\mu)$	ER reference	ΔG_{exp}
0002eth	ethane	1.70	2.6	J. Chem. Phys. 2002, 117 (8): 3605-3616.	1.83
0217wat	water	-4.64	-8.2		-6.31
0053phe	Phenol	-5.96	-6.0	AIP Conf. Proc. 2007, 963 (2), 342-345.	-6.62
0035ben	benzene	-1.45	0.0		-0.87
0001met	methane	1.97	1.9	Chem. Phys. Lett. 2010, 496 (4), 351-355.	2
0044met	methanol	-4.29	-5.2		-5.11
0045eth	ethanol	-4.60	-4.8		-5.01
0003pro	propane	1.31	2.8		1.96
0004nbu	n-butane	1.37	3.0		2.08
0036tol	toluene	-1.06	-0.1		-0.89
0136met	methanethiol	0.78	-0.7		-1.24
0233ethb	acetamide	-10.76	-9.2		-9.71
0057pcr	p-cresol	-5.56	-5.2		-6.14
0068ani	anisole	-2.41	-1.8	J. Chem. Theory Comput. 2015, 11 (3), 1181-1194.	-2.45
0021eth	ethene	0.88	1.9		1.27
0023str	s-trans-1,3-butadiene	0.01	1.5		0.61
	Pearson coefficient	0.97	0.99		
	MAE	0.66	0.70		
	RMSE	0.84	0.81		
